# Hepatic transcriptomic profiling reveals early toxicological mechanisms of uranium in Atlantic salmon (*Salmo salar*)

**DOI:** 10.1186/1471-2164-15-694

**Published:** 2014-08-20

**Authors:** You Song, Brit Salbu, Hans-Christian Teien, Lene Sørlie Heier, Bjørn Olav Rosseland, Tore Høgåsen, Knut Erik Tollefsen

**Affiliations:** Department of Environmental Sciences (IMV), Faculty of Environmental Science and Technology, Centre for Environmental Radioactivity (CERAD), Norwegian University of Life Sciences (NMBU), P.O. Box 5003, N-1432 Ås, Norway; Norwegian Institute for Water Research (NIVA), Gaustadalléen 21, N-0349 Oslo, Norway; Department of Ecology and Natural Resource Management, Faculty of Environmental Science and Technology, Norwegian University of Life Sciences (NMBU), P.O. Box 5003, N-1432 Ås, Norway

**Keywords:** Depleted uranium, Fish, *in vivo*, Microarray, Transcription, Pathway, Mode of action, Toxicological mechanism

## Abstract

**Background:**

Uranium (U) is a naturally occurring radionuclide that has been found in the aquatic environment due to anthropogenic activities. Exposure to U may pose risk to aquatic organisms due to its radiological and chemical toxicity. The present study aimed to characterize the chemical toxicity of U in Atlantic salmon (*Salmo salar*) using depleted uranium (DU) as a test model. The fish were exposed to three environmentally relevant concentrations of DU (0.25, 0.5 and 1.0 mg U/L) for 48 h. Hepatic transcriptional responses were studied using microarrays in combination with quantitative real-time reverse transcription polymerase chain reaction (qPCR). Plasma variables and chromosomal damages were also studied to link transcriptional responses to potential physiological changes at higher levels.

**Results:**

The microarray gene expression analysis identified 847, 891 and 766 differentially expressed genes (DEGs) in the liver of salmon after 48 h exposure to 0.25, 0.5 and 1.0 mg/L DU, respectively. These DEGs were associated with known gene ontology functions such as generation of precursor metabolites and energy, carbohydrate metabolic process and cellular homeostasis. The salmon DEGs were then mapped to mammalian orthologs and subjected to protein-protein network and pathway analysis. The results showed that various toxicity pathways involved in mitochondrial functions, oxidative stress, nuclear receptor signaling, organ damage were commonly affected by all DU concentrations. Eight genes representative of several key pathways were further verified using qPCR No significant formation of micronuclei in the red blood cells or alterations of plasma stress variables were identified.

**Conclusion:**

The current study suggested that the mitochondrion may be a key target of U chemical toxicity in salmon. The induction of oxidative stress and uncoupling of oxidative phosphorylation may be two potential modes of action (MoA) of DU. These MoAs may subsequently lead to downstream events such as apoptosis, DNA repair, hypoxia signaling and immune response. The early toxicological mechanisms of U chemical toxicity in salmon has for the first time been systematically profiled. However, no other physiological changes were observed. Future efforts to link transcriptional responses to adverse effects have been outlined as important for understanding of potential risk to aquatic organisms.

**Electronic supplementary material:**

The online version of this article (doi:10.1186/1471-2164-15-694) contains supplementary material, which is available to authorized users.

## Background

Uranium (U) is a naturally occurring heavy metal of the actinide series and decays by emitting alpha particles, thus exhibiting both chemical and radiological toxicity. Due to its ability to undergo fission and liberate energy, U is commonly used as fuel for nuclear reactors or for military weapon purposes. Uranium is usually released anthropogenically to the aquatic environment through the nuclear fuel cycle, such as U mill tailings, mill and refining, effluent from conversion plants, and stack emissions [1]. Especially near the U mill tailings, U dust particles can be easily washed out by precipitation into surface water. The U concentration in surface water may range from 0.02 μg/L to 3 mg/L, depending on the geological conditions [2–4]. Uranium may accumulate in bone, liver and kidney of an organism, but may not be biomagnified [5]. In fish, such as Atlantic salmon and zebrafish (*Danio rerio*), U has been shown to accumulate in gill, liver, brain and skeletal muscles [6, 7]. Due to the chemical and radioactive properties, U as a single substance may produce multiple-stressor effects in an organism, thus complicating the subsequent hazard assessment.

Natural U has relatively low radioactivity and usually goes through enrichment processes to obtain higher fraction (%) of the radioactive isotope ^235^U. The remaining material mainly contains ^238^U and is referred to as depleted uranium (DU). Depleted U has low specific activity (approximately 1.47 × 10^4^ Bq/g), but exhibits identical chemical properties as natural U. Depleted U is also widely used in many military and civilian applications such as armor and armor penetrators, counterweights for aircraft construction and irradiation shielding [8]. Research on enriched and depleted U showed that the genotoxicity of U may be dependent on its isotopic composition [9]. However, even for radioactive natural U, its chemical toxicity may still pose greater risk than its radiological toxicity [10]. These properties along with the fact that many types of commercially available U (e.g. U nitrate, U acetate) are made from DU suggests that DU is a good model to study the effects and mechanisms of natural U.

The toxic effects of DU, such as neurotoxicity, DNA damage and carcinogenicity, reproductive toxicity, immunosuppression and organ/tissue toxicity have been well documented for mammalian species [11]. Similar effects have also been reported for other organisms, albeit the toxicological mechanisms of DU are rather complex and have not been as well characterized in fish [5]. Several studies have reported that increase in reactive oxygen species (ROS) production by DU may cause oxidative stress as a main toxicological modes of action (MoA) and modulate various cellular responses such as activation of antioxidant defense system, DNA damage and repair, programmed cell death (apoptosis), enhanced protein degradation and inflammation, stimulation of the immune system, altered mitochondrial metabolism and ion transport, modulation of signal transduction and catabolism [6, 7, 12, 13]. However, since these studies were performed based on the assessment of predefined genes or protein expression response as biomarkers for exposure and effects, they may not fully address the complex cellular responses and toxicological pathways being perturbed when exposed to DU. Genome-wide genomic tools such as DNA microarrays, next generation sequencing and Serial Analysis of Gene Expression (SAGE) analysis, may facilitate unbiased assessment of the MoA and in-depth characterization of the toxicological mechanisms of DU. Results from such broad-content approaches may potentially provide links between interactions with the biological (toxicological) targets, perturbations of key cellular events and adverse effects, thus aiding the development of adverse outcome pathways (AOPs) for impact on organisms and/or the population health [14].

The present study was carried out as an expansion of a previous reported experiment [7] to characterize the early hepatic toxicological mechanisms of U chemical toxicity in fish. The study was conducted with Atlantic salmon (*Salmo salar*), an ecological and economically important fish species in the temperate areas of Europe, using a high-density 60,000-feature (60 k) salmonid oligoarray. The objectives of the present study were: 1) to profile the early hepatic transcriptional responses after environmentally relevant DU exposure; 2) to determine the concentration-response relationship of DU in Atlantic salmon; 3) to characterize the potential MoAs of U.

## Results

### Exposure conditions

The exposure conditions and U concentrations have been reported previously [7]. Briefly, the exposure media was characterized by conductivity 4.4 ± 1.0 mS/m, pH 7.1-7.3, temperature 4.4 ± 0.2°C, and total organic carbon (TOC) 4.6 ± 0.6 mg/L throughout the experiment. No significant changes of water quality variables were observed. The actual U concentration prior to exposure were 0.26 mg/L (nominal: 0.25 mg/L), 0.53 mg/L (nominal: 0.5 mg/L) and 1.0 mg/L (nominal: 1.0 mg/L). As no large differences were observed between nominal and actual U concentrations, the treatment groups will be referred to in the text as their nominal concentrations. A total of 38–74% U was found to be low molecular mass (LMM), presumably uranyl species. Liver concentrations (n = 3) of U were 2.9 ± 1.5 ng/g ww, 4.9 ± 1.9 ng/g ww and 7.5 ± 2.1 ng/g ww in the nominal group 0.25 mg U/L, 0.5 mg U/L and 1.0 mg U/L, respectively. No U was detected (detection limit = 3 SD above background) in either exposure water or in liver for control group (data not shown). No fish died or developed observable external morphological changes.

### Global gene expression

**Figure 1:**
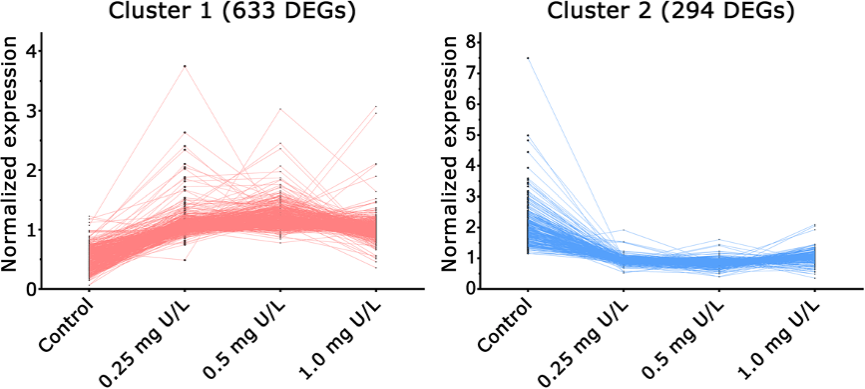
**K-means clustering of global gene expression.** A K-means clustering analysis showing the two major patterns of global gene expression responses in the liver of Atlantic salmon (*Salmo salar*) after 48 h waterborne exposure to 0.25. 0.5 and 1.0 mg/L nominal concentrations of depleted uranium (DU).

Liver tissues of exposed fish were used to obtain information of transcriptomic response to U exposure. In total 927 gene transcripts with fold change ≥ 1.5 were found to be differentially expressed (one-way ANOVA) after 48 h U exposure. To determine the concentration-response relationship between U exposure and global gene expression, the differentially expressed genes (DEGs) were clustered according to their responses. Figure 1 shows the two major patterns of transcriptomic response to different U concentrations. A general tendency of increased transcriptional responses from low (0.25 mg/L) to medium U exposure (0.5 mg/L), and decreased from medium to high U exposure (1.0 mg/L) was observed.

**Figure 2:**
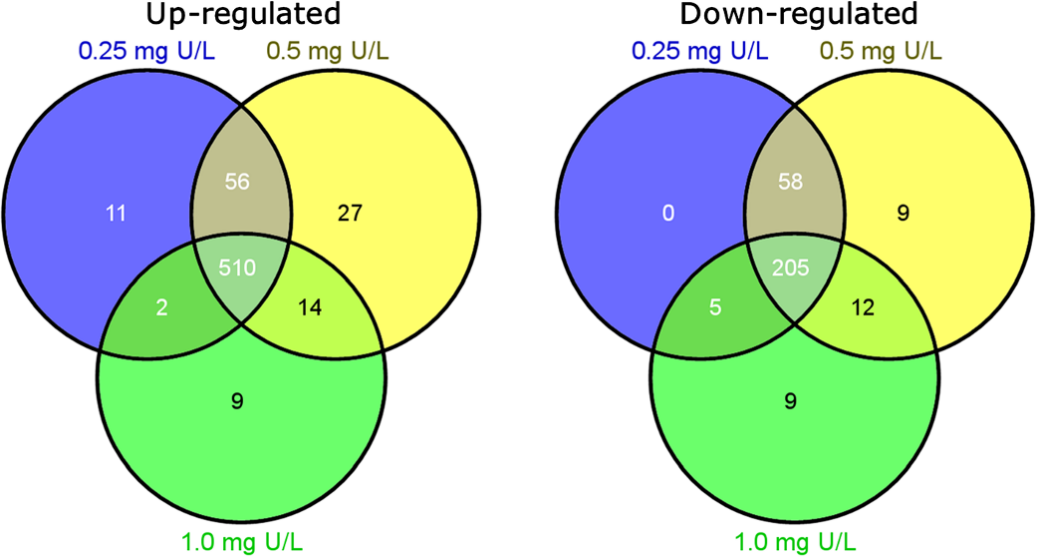
**Differentially expressed gene transcripts (DEGs).** A Venn diagram analysis showing an overview of common and unique DEGs that were differentially regulated (FC ≥ 1.5) in the liver of Atlantic salmon (*Salmo salar*) after 48 h waterborne exposure to 0.25, 0.5 and 1.0 mg/L nominal concentrations of depleted uranium (DU).

To further identify the concentration-related DEGs, a Tukey HSD posthoc test was performed on genes determined to be significant by the ANOVA test. In total 847 (579 up- and 268 down-regulated), 891 (607 up- and 284 down-regulated), and 766 (535 up- and 231 down-regulated) hepatic gene transcripts with absolute fold change ≥ 1.5 were found to be differentially expressed in Atlantic salmon after exposure to 0.25 (low), 0.5 (medium) and 1.0 mg/L (high) U, respectively. More up-regulated genes than down-regulated genes were found in all treatment groups. A Venn diagram analysis was performed to separate common and unique DEGs that were regulated by different U concentrations. The results (Figure 2) clearly showed that the majority of DEGs were commonly regulated by all treatments and only a few ones were found to be concentration-specific. Among the up-regulated DEGs, 510 were commonly regulated by all treatments, 11 were uniquely regulated by low U, 27 were uniquely regulated by medium U and 9 were regulated by high U treatment. Among the down-regulated DEGs, 205 were commonly regulated by all treatments, no DEG was specifically regulated by low U, 9 were uniquely regulated by medium U and 9 were regulated by high U treatment. The low and medium U groups tended to have more common genes than that between medium and high U treatments, or between low and high U treatments. A complete list of DEGs can be found in Additional file 1: Table S1.

### Functional enrichment analysis

**Figure 3:**
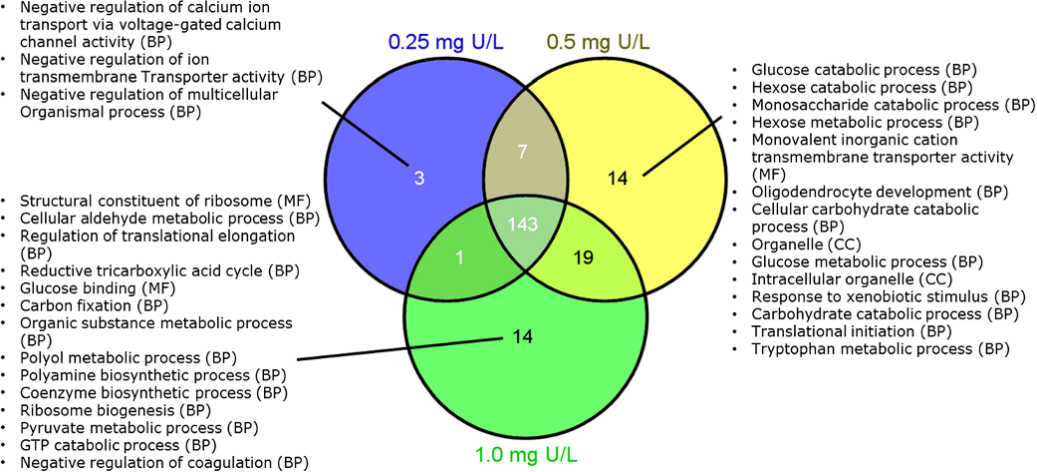
**Gene Ontology (GO) functions.** A Venn diagram analysis of GO terms that were significantly overrepresented (p < 0.05) in the liver of Atlantic salmon (*Salmo salar*) after 48 h waterborne exposure to 0.25, 0.5 and 1.0 mg/L nominal concentrations of depleted uranium (DU). The results were related to up-regulated genes. Lists of descriptions were corresponding to the GOs that were uniquely regulated by different concentrations of U. BP: biological process; MF: molecular function; CC: cellular component.

In total 154, 183 and 177 GO terms related to up-regulated DEGs were found to be significantly overrepresented after exposure to low, medium and high U treatments, respectively. No significant enrichment of GO was found based on down-regulated DEGs. The Venn diagram analysis (Figure 3) showed that the majority (413 terms) of the GO biological functions was commonly regulated by all U concentrations and only a few functions were found to be treatment-specific. The biological functions uniquely regulated by low U treatment were mainly related to ion transport and multicellular organismal regulation. Uniquely overrepresented GO functions following medium U exposure were mainly associated with cellular metabolic and catabolic processes, transporter activity, translation and general stress response. Exposure to high U led to unique regulation of DEGs with biological functions such as nutrient metabolic processes, translation and coagulation.

**Figure 4:**
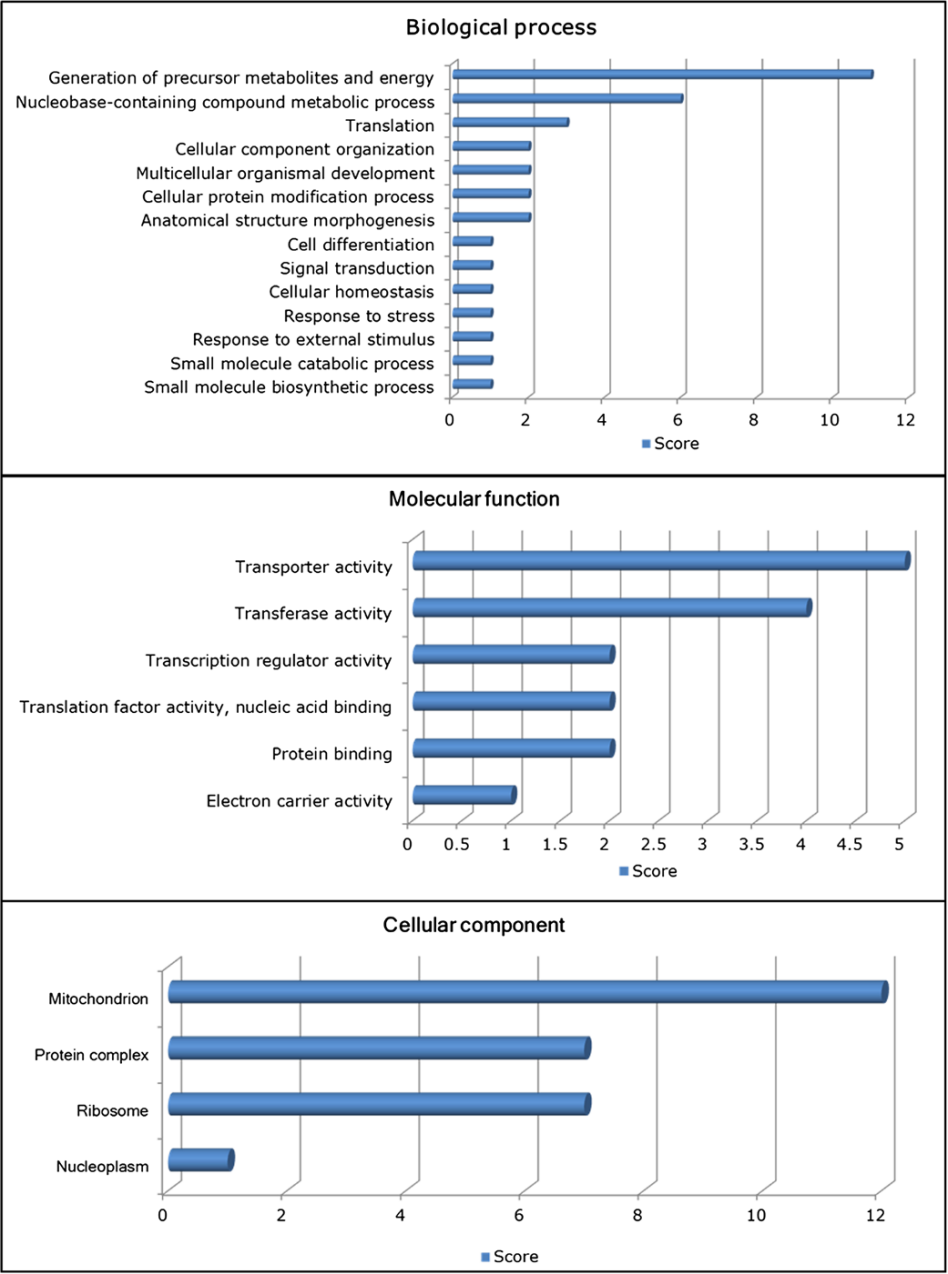
**Common biological functions regulated by all concentrations of depleted uranium.** An overview of overrepresented gene ontology (GO) biological processes, molecular functions and cellular component that were commonly regulated in the liver of Atlantic salmon (*Salmo salar*) after 48 h waterborne exposure to 0.25, 0.5 and 1.0 mg/L nominal concentrations of depleted uranium (DU). Score = number of supporting GO terms within the same directed acyclic relationship (i.e. functional category).

Enriched GO functions that were commonly regulated by all U concentrations were shown in Figure 4. Several biological processes such as generation of precursor metabolites and energy, nucleobase-containing compound metabolic process and carbohydrate metabolic process were found to be the top functions affected by U. Other processes directly related to U-induced stress responses such as signal transduction, cellular homeostasis, cellular differentiation, response to stress and response to external stimulus were identified. The up-regulated DEGs had important molecular functions such as transporter activity, transferase activity and translation factor activity (nucleic acid binding). A number of DEGs were also found to be associated with cellular activities localized in the mitochondrion, protein complex and ribosome, whereas the nucleoplasm was less affected. A complete list of overrepresented GOs can be found in Additional file 1: Table S2.

### Gene network and pathway analyses

Protein-protein interaction-based gene network and pathway analyses were performed using mapped salmon DEGs towards mammalian orthologs to get more insight into the toxicological mechanisms of U. In total 67.9% (low U), 67.7% (medium U) and 69% (high U) DEGs were successfully mapped (Additional file 1: Table S3). Network and pathway analyses were performed using either complete DEG lists or two separated lists of DEGs representing up- and down-regulation of genes. Better enrichment of DEGs in their supporting pathways was observed when using both up- and down-regulated DEGs rather than separated lists. Table 1 shows the top gene networks that were regulated by U. The gene networks that were by different U concentrations had common functions, such as hematological disease, hereditary disorder, tissue morphology, lipid metabolism, cell death and survival and DNA repair.

**Table 1:** **Top gene networks (score > 1, focus molecules > 1) induced in the liver of Atlantic salmon (** ***Salmo salar*** **) after 48 h waterborne exposure to 0.25, 0.5 and 1.0 mg/L nominal concentrations of depleted uranium (DU)**

Top network functions	0.25 mg U/L		0.5 mg U/L		1.0 mg U/L	
	Score	DEGs	Score	DEGs	Score	DEGs
Hematological Disease, Hereditary Disorder, Tissue Morphology	181	123	181	123	181	123
Cellular Assembly and Organization, Cellular Function and Maintenance, Cell Morphology	110	89	110	89	110	89
Hereditary Disorder, Metabolic Disease, Lipid Metabolism	81	70	81	70	81	70
Developmental Disorder, Hereditary Disorder, Metabolic Disease	78	68	79	69	79	69
Endocrine System Development and Function, Small Molecule Biochemistry, Drug Metabolism	70	64	70	64	70	64
Cell Death and Survival, Cellular Movement, Tumor Morphology, Hair and Skin Development and Function	57	56	58	57	58	57
DNA Replication, Recombination, and Repair, Energy Production, Nucleic Acid Metabolism	54	54	53	53	54	54
Protein Synthesis, Gene Expression, Developmental Disorder	13	22	13	22	13	22

**Figure 5:**
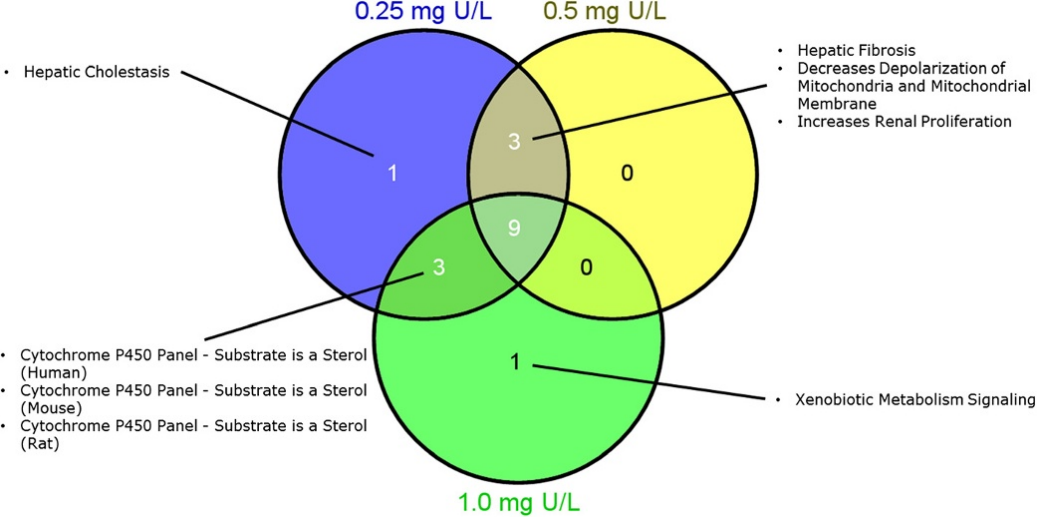
**Common and unique toxicity pathways between different uranium concentrations.** A Venn diagram showing common and unique toxicity pathways that were regulated in the liver of Atlantic salmon (*Salmo salar*) after 48 h waterborne exposure to 0.25, 0.5 and 1.0 mg/L nominal concentrations of depleted uranium (DU).

**Figure 6:**
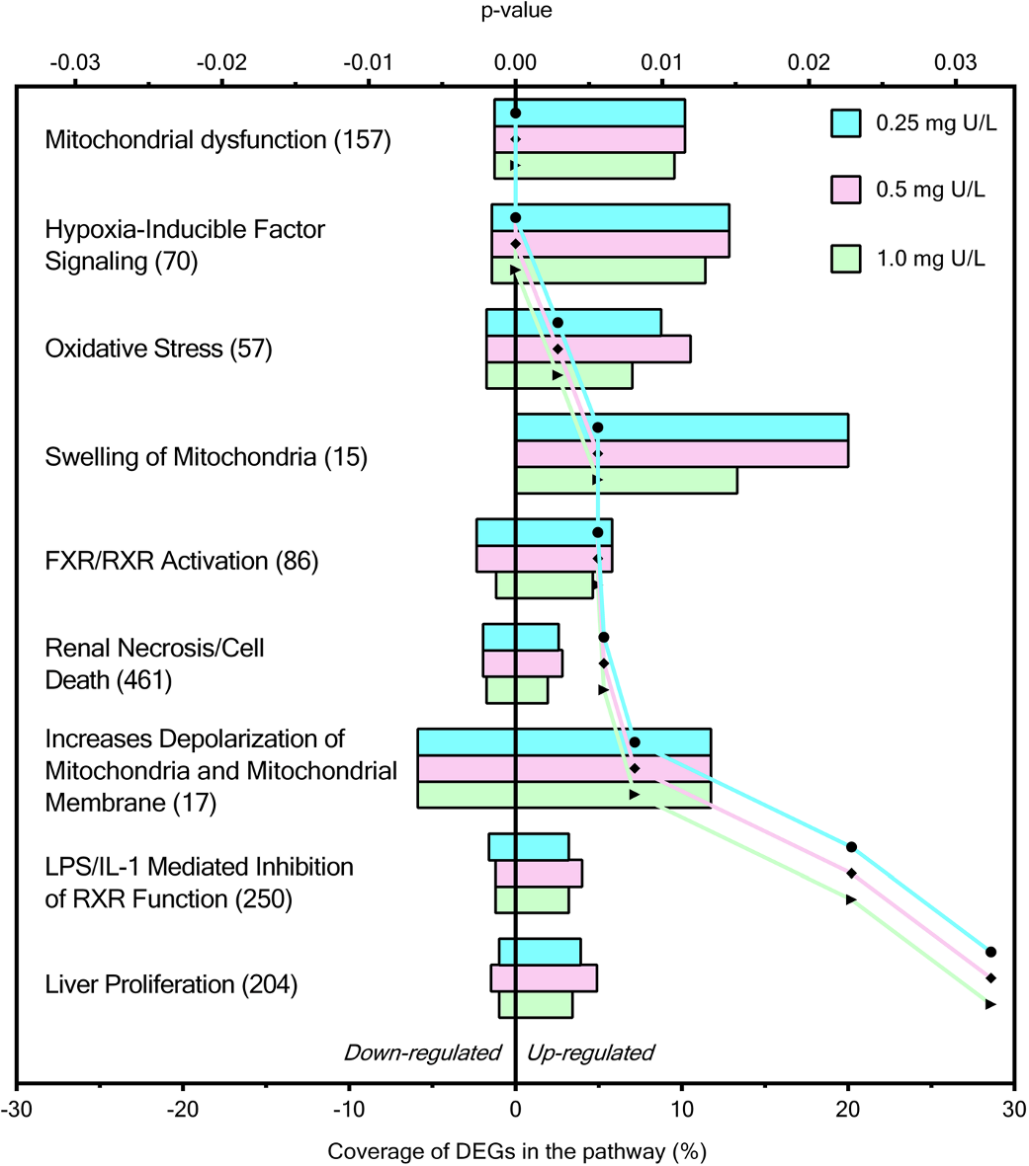
**Commonly regulated toxicity pathways and supporting differentially expressed genes (DEGs) among all uranium concentrations.** Toxicity pathways and number of supporting differentially expressed genes (DEGs) that were commonly regulated in the liver of Atlantic salmon (*Salmo salar*) after 48 h waterborne exposure to 0.25, 0.5, 1.0 mg/L nominal concentrations of depleted uranium (DU). Bars indicate the percentages of up- or down-regulated DEGs found in the present study compared to the total number of supporting genes in the pathway (given in parenthesis). Dotted lines indicate the patterns of statistical significance across different pathways.

Pathway analyses further identified sets of DEGs that were involved in specific biological or toxicological functions. A Venn diagram analysis was performed first to identify common and unique toxicity pathways that were affected by different U concentrations (Figure 5). Nine toxicity pathways (Figure 6) were found to be commonly regulated by all U concentrations, including pathways related to mitochondrial functions (mitochondrial dysfunction, swelling of mitochondria, increases depolarization of mitochondria and mitochondrial membrane), stress responses to ROS (oxidative stress, hypoxia-inducible factor signaling), nuclear receptor signaling (farnesoid X receptor (FXR)/ retinoid X receptor (RXR) activation, endotoxin lipopolysaccharide (LPS)/interleukin-1 (IL-1) mediated inhibition of RXR function) and organ damage (renal necrosis/cell death, liver proliferation). Three toxicity pathways were commonly regulated by low and medium U, including pathways related to mitochondrial functions (decreases depolarization of mitochondria and mitochondrial membrane) and organ damage (hepatic fibrosis, increases renal proliferation). The biotransformation-related pathways (cytochrome P450 panel-substrate is a sterol) derived from different species were commonly regulated by low and high U concentrations. Toxicity pathways related to potential organ damage (hepatic cholestasis) and biotransformation (xenobiotic metabolism signaling) were uniquely regulated by low and high U, respectively. No toxicity pathway was found to be uniquely regulated by medium U. The ratios between supporting DEGs identified in this study and the putative total supporting genes in a toxicity pathway ranged from 0.04 to 0.2. A complete list of significantly enriched toxicity pathways can be found in Additional file 1: Table S4).

**Figure 7:**
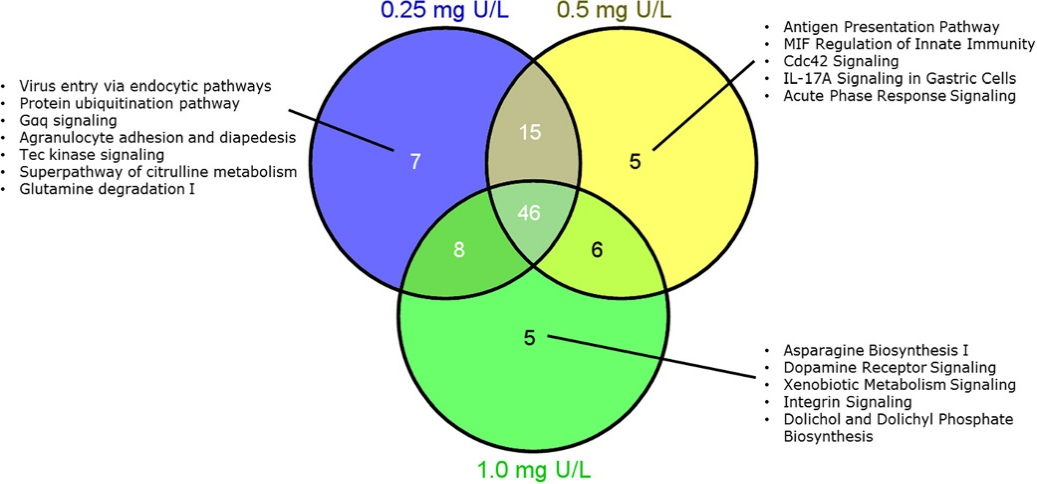
**Common and unique canonical pathways between different uranium concentrations.** A Venn diagram showing the common and unique canonical pathways that were regulated in the liver of Atlantic salmon (*Salmo salar*) after 48 h waterborne exposure to 0.25, 0.5 and 1.0 mg/L nominal concentrations of depleted uranium (DU).

Canonical pathways were also studied to get more insight into the toxicological mechanisms of U. In total 76, 72 and 65 canonical pathways were found to be significantly affected by low, medium and high concentration of U, respectively. A Venn diagram analysis was also performed to identify common and unique canonical pathways between different U concentrations (Figure 7). Among all enriched canonical pathways, 7 were uniquely affected by low U, 5 uniquely affected by medium U and 5 uniquely affected by high U treatment. There were 46 pathways being commonly affected by all U concentrations. These pathways were mainly grouped into 10 apical functional categories (Table 2), including amino acids degradation, cell growth and development, energy-related cellular metabolic process, cellular immune response, free radical scavenging and apoptosis, hematological response, intracellular signal transduction, nervous system signaling, nuclear receptor signaling and regulation of cell cycle, DNA replication, repair and transcription. A complete list of significantly enriched canonical pathways can be found in Additional file 1: Table S5.

**Table 2:** **Canonical pathways and supporting differentially expressed genes (DEGs) that were commonly regulated by 0.25, 0.5 and 1.0 mg/L nominal concentrations of depleted uranium (DU)**

Apical toxicological category	Ingenuity canonical pathways	Supporting DEGs
Amino acids degradation	Tryptophan Degradation to 2-amino-3-carboxymuconate Semialdehyde	KMO↑,TDO2↑
	Valine Degradation I	DLD↑,ALDH6A1↑,BCKDHB↑
Cell growth and development	Epithelial Adherens Junction Signaling	MET↑,CDH1↓,MYL6↑,ARPC5L↑,MYH7↑,TCF7L1↓,CDC42↑,ACTG1↓,TUBA1B↑
	Sertoli Cell-Sertoli Cell Junction Signaling	MAP2K4↑,CDH1↓,MAPK14↓,PPAP2B↑,CLDN18↓,IGSF5↑,CDC42↑,ACTG1↓,TUBA1B↑
	Germ Cell-Sertoli Cell Junction Signaling	MAP2K4↑,CDH1↓,MAPK14↓,RHOB↑,PPAP2B↑,CDC42↑,RHOF↑,ACTG1↓,TUBA1B↑,FNBP1↑
	Remodeling of Epithelial Adherens Junctions	MET↑,DNM1↓,CDH1↓,ARPC5L↑,ACTG1↓,TUBA1B↑
	ILK Signaling	MAP2K4↑,CDH1↓,FN1↓,MYL6↑,RHOB↑,PPAP2B↑,MYH7↑,CDC42↑,RHOF↑,ACTG1↓,FNBP1↑,DSP↓
	Actin Cytoskeleton Signaling	FN1↓,MYL6↑,ARPC5L↑,PIKFYVE↑,RDX↓,MYH7↑,CDC42↑,GIT1↓,TTN↓,ACTG1↓
Cellular immune response	HMGB1 Signaling	MAP2K4↑,MAPK14↓,RHOB↑,CDC42↑,RHOF↑,FNBP1↑
	IL-22 Signaling	MAP2K4↑,JAK1↓,MAPK14↓
Free radical scavenging and apoptosis	Mitochondrial Dysfunction	MAP2K4↑,SDHA↑,COX6B1↑,CPT1A↓,UQCR11↑,SDHC↑,COX7A2L↑,DHODH↑,MAOB↓,PRDX3↑,TXN2↑,UQCRFS1↑,NDUFS2↑,SDHD↑,NDUFA3↑,AIFM1↑,COX15↑
	NAD biosynthesis II (from tryptophan)	KMO↑,TDO2↑,QPRT↑
	Tight Junction Signaling	MYL6↑,RAB13↑,CLDN18↓,IGSF5↑,MYH7↑,CDC42↑,ACTG1↓,CSTF3↑
Hematological response	Hypoxia Signaling in the Cardiovascular System	TP53↑,P4HB↑,HSP90AB1↑,SUMO1↑,NQO1↑,UBE2D4↓,UBE2E1↑
	Inhibition of Angiogenesis by TSP1	MAP2K4↑,HSPG2↑,TP53↑,MAPK14↓
	Intrinsic Prothrombin Activation Pathway	F10↑,F9↑,COL18A1↓
	Coagulation System	F10↑,F9↑,F7↑,SERPINF2↑,SERPIND1↑
	Role of JAK family kinases in IL-6-type Cytokine Signaling	MAP2K4↑,JAK1↓,MAPK14↓
Intracellular signal transduction	Signaling by Rho Family GTPases	MAP2K4↑,CDH1↓,RHOB↑,MYL6↑,ARPC5L↑,GNB2L1↑,SEPT7↑,RDX↓,PIKFYVE↑,RHOF↑,CDC42↑,ACTG1↓,FNBP1↑
	Actin Nucleation by ARP-WASP Complex	RHOB↑,ARPC5L↑,CDC42↑,RHOF↑,FNBP1↑
	RhoA Signaling	MYL6↑,ARPC5L↑,SEPT7↓,PIKFYVE↑,RDX↓,TTN↓,ACTG1↓
	Glucocorticoid Receptor Signaling	MAP2K4↑,GTF2A2↑,POLR2F↑,JAK1↓,MAPK14↓,HSP90AB1↑,SUMO1↑,GTF2E2↑,HLTF↑,POLR2I↑,TAF13↑
	RhoGDI Signaling	CDH1↓,MYL6↑,RHOB↑,ARPC5L↑,GNB2L1↑,PIKFYVE↑,RDX↓,CDC42↑,RHOF↑,ACTG1↓,FNBP1↑

Full descriptions of gene symbols can be found in Additional file 1: Table S3. Arrow sign ↑ indicates up-regulation, ↓ indicates down-regulation.

### Quantitative real-time rtPCR verification

**Figure 8:**
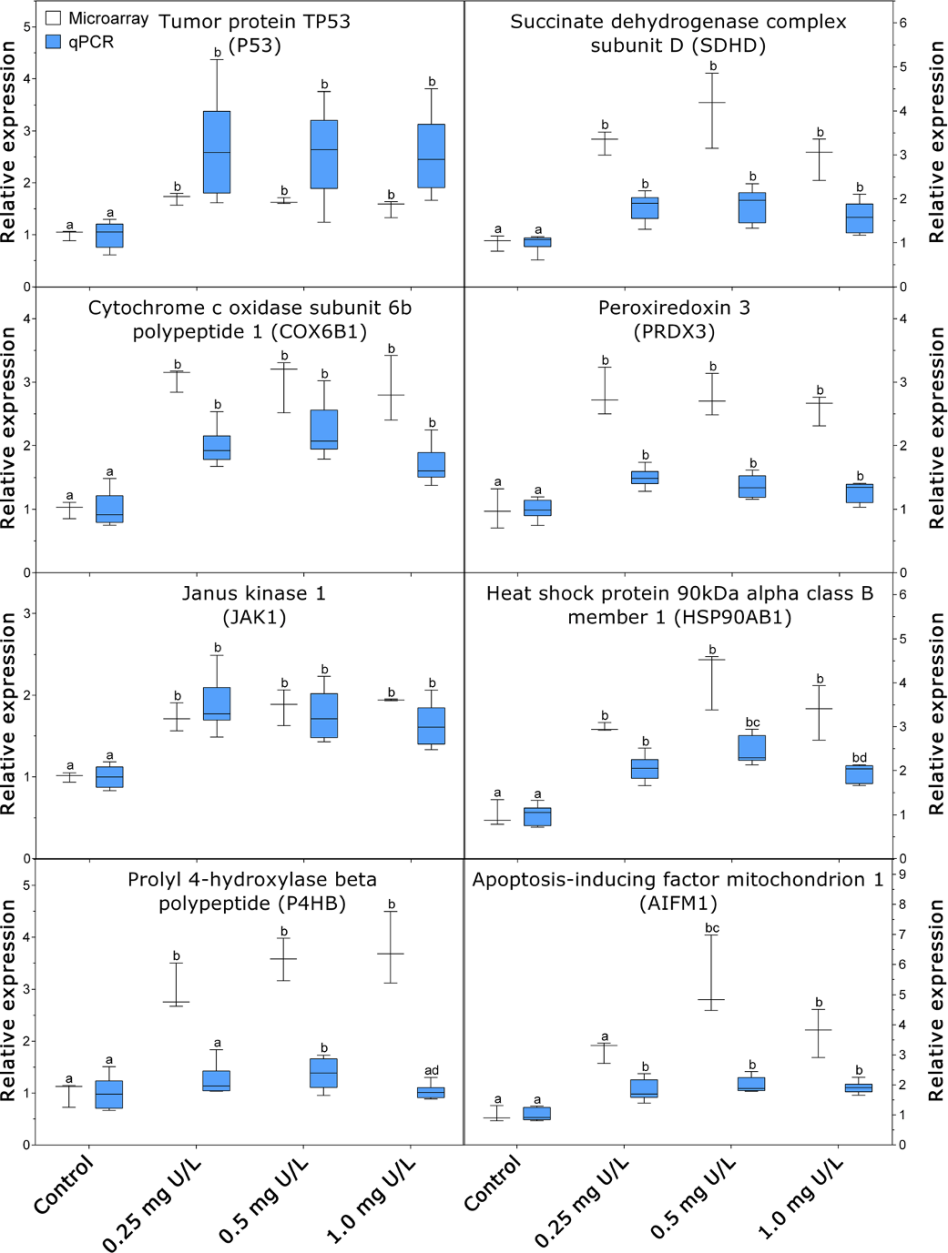
**Quantitative real-time rtPCR (qPCR) verification of biomarker gene response.** A comparison of biomarker gene expressions measured by microarray (N = 3) and qPCR (N = 6) after 48 h waterborne exposure to 0.25, 0.5 and 1.0 mg/L nominal concentrations of depleted uranium (DU) in the liver of Atlantic salmon (*Salmo salar*). The lower and upper edge of box represent 25% median and 75% median of data, respectively, the middle line in the box represents the data median, the whiskers represent the data range (from the min. to max.). Left column: results from qPCR analysis; Right column: results from microarray analysis. a: not significantly different from the control; b: significantly different from the control; c: significantly different from 0.25 mg U/L treatment; d: significantly different from 0.5 mg U/L treatment.

Quantitative real-time rtPCR was performed to verify 8 selected DEGs involved in a few important pathways found by microarray analysis, including apoptosis-inducing factor mitochondrion-associated 1 (AIFM1), janus kinase 1 (JAK1), heat shock protein 90 kDa alpha class B member 1 (HSP90AB1), succinate dehydrogenase complex subunit D (SDHD), prolyl 4-hydroxylase beta polypeptide (P4HB), cytochrome c oxidase subunit VIb polypeptide 1 (COX6B1), peroxiredoxin 3 (PRDX3) and tumor protein p53 (P53). Significant induction of these genes was found by qPCR analysis (Figure 8). A general tendency of increased expression from low to medium U, and decreased expression from medium to high U concentration was found for most of the target genes tested. Compared to the microarray results, some differences were found in the magnitude of gene expression. All genes induced by U, except for P53, had relatively larger fold changes measured by microarray than that measured by qPCR.

### Plasma variables and micronucleus assay

The blood samples were also collected in addition to liver to determine potential physiological changes after exposure to U. As reported previously [7], no significant alterations of plasma glucose, Na, K or hematocrit were found. A slight increase of micronuclei formation was observed in the red blood cells in 0.25 mg /L DU exposed fish. However, the difference was significant from the control [7].

## Discussion

### Uranium bioaccumulation

As reported previously, U was found to accumulate in the liver of Atlantic salmon after 48 h exposure and the liver concentrations increased with increasing the water concentrations of U [7]. The bioaccumulation of U in fish, especially in gill, liver and kidney, has been well documented by a number of studies [6, 7, 15–20]. The internal concentrations of U in different tissues are likely dependent on U speciation in the test water, concentrations of competing ions and toxicokinetics in fish. The observed effects after 48 h waterborne exposure to 0.25, 0.5 and 1.0 mg/L nominal concentrations of DU is therefore likely due to the U uptake and U liver concentration (2.9 ± 1.5 ng/g ww, 4.9 ± 1.9 ng/g ww and 7.5 ± 2.1 ng/g ww) and not to the total U concentration in water.

### Early transcriptional responses to uranium

The present study focused on a combination of classical statistical treatment and functional enrichment analysis followed by customized identification of potential protein-protein interactions, toxicity and canonical pathways on the basis of mapping to mammalian orthologs. The protein-protein interactions, toxicity and canonical pathway analyses were considered to be exploratory, as the mammalian pathways may not always represent the same functions as that in fish. However, the correspondence between the present data and existing knowledge on stress responses in fish after U exposure provide confidence that the approach chosen herein may serve as a platform to guide future investigations. The major findings will be summarized and discussed below.

#### Mitochondrion electron transport chain

The analysis of global transcriptional changes and associated functional interpretation in the present study suggested that the mitochondrion, which plays a central role in the production of adenosine-5’-triphosphate (ATP) in oxidative phosphorylation (OXPHOS), may be one of the main targets of U hepatotoxicity in Atlantic salmon after short-term exposure. This was first revealed by GO-based functional analysis of DEGs associated with the mitochondrion as a cellular target, and further confirmed by pathway analyses where mitochondrial dysfunction, increases depolarization of mitochondria and mitochondrial membrane, and swelling of mitochondria were found to be affected by all U concentrations. The mitochondrial dysfunction pathway (Additional file 2: Figure S1) showed that several encoding genes of major protein complexes in the mitochondrial electron transport chain (ETC), such as NADH dehydrogenase (ubiquinone) Fe-S protein 2 (NDUFS2, Complex I), succinate dehydrogenase complex subunit D (SDHD, complex II), ubiquinol-cytochrome c reductase (UQCR11, complex III) and cytochrome c oxidase subunit VI b polypeptide 1 (COX6B1, complex IV), were found to be up-regulated, indicating potentially elevated ETC activity after U exposure. The pathway analysis did not identify genes in the complex V being affected, probably due to the lack of successful mapping of salmon DEGs towards mammalian orthologs. Indeed, two unmapped salmon DEGs which have important functions in complex V were induced by U, including ATP synthase H^+^ transporting mitochondrial F0 complex subunit F6 (ATP5J), which was regulated by all U concentrations, and complex V subunit ATP synthase H^+^ transporting mitochondrial F1 complex beta polypeptide (ATP5B), which was regulated by medium U treatment. These findings supported the hypothesis that core components of the mitochondrial ETC were likely affected by U. The mitochondrion ETC activity and associated ATP synthesis may have been modulated by U after 48 h exposure. This was also supported by the result from GO-based functional analysis that generation of precursor metabolites and energy was found to be the top overrepresented GO biological process affected by U. In addition, the eukaryotic citric acid (TCA) cycle II as a main part of the nutrient metabolic processes in the aerobic respiration was identified to be one of the highly enriched canonical pathways, indicating potential effects of U on energy metabolism in fish. These findings were in agreement with that reported by a few previous fish studies. Work by Lerebours and co-workers [6] reported that genes involved in the mitochondrial metabolism such as cytochrome c oxidase subunit I (COX-I) and mitochondrial ATP synthase subunit b (ATP5f1) were induced by low concentrations (23 and 130 μg/L) of U during a 28 d exposure in zebrafish. Lerebours and colleagues [12] further measured the mitochondrial energetic metabolism in the skeletal muscles and brain of zebrafish exposed to 30 and 100 μg/L of U for 28 d and found that the basal respiration rate was increased in the brain at day 10 and in the muscles at day 28 with a few proteins involved in the ETC being up-regulated, such as COX-IV (brain) and COX-I (muscle).

The mitochondrial membrane potential (MMP), a driving force for the operation of ATP synthase (complex V) to produce ATP, is dependent on the ETC activity [21]. The present study identified two toxicity pathways involved in the depolarization of mitochondria and mitochondrial membrane. One of them was related to the increase in the MMP and the other related to the reduction of the MMP. These toxicity pathways were supported by different sets of DEGs, probably indicating the activation of multiple mechanisms related to potentially decreased MMP after U exposure. In fact, dissipation of MMP by U has been reported by a number of studies in mammals previously [22–26] and may also be a key mechanism of U chemical toxicity in fish. A study by Pourahmad and co-workers [27] found that the collapse of MMP was correlated with increased mitochondrial permeability transition (MPT) in rat hepatocyte due to the opening of mitochondrial permeability transition pore (MPTP) after U exposure. The opening of MPTP may be a consequence of the oxidation of thiol groups by U-induced ROS in the MPTP region in the mitochondrial inner membrane [27], or as a result of perturbed mitochondrial osmolarity, such as the alteration of intra-mitochondrial calcium level [12, 28, 29]. It has also been suggested that uranyl ions may form stable complexes with ATP by binding to the phosphate groups [30], thus influencing the synthesis of ATP and its cross-membrane translocation. Interestingly, several DEGs involved in these processes, such as the calcium channel voltage-dependent L type alpha 1C subunit (CACNA1C) and sodium/potassium transporting ATPase subunit beta-3 (ATP1B3) related to the calcium transportation, and adenosine diphosphate (ADP)/ATP translocase 2 (SLC25A5) related to the ADP/ATP cross-membrane transportation were found to be regulated following U exposure. The differential expression of these transporter genes may be potentially caused by disturbance of calcium and ATP homeostasis in the cells by U. Taken together, U may induce ROS, increase the ion concentration and/or interfere with ATP/ADP molecules in the mitochondria, thus causing an increase in the inner membrane MPT and dissipation of the proton gradient (i.e. MMP) by calcium overload and/or U ion accumulation on the mitochondrial inner membrane. The activation of ETC genes may therefore be a compensatory mechanism to re-establish the MMP, adjust the osmolarity in the mitochondria and overall secure the ATP production.

#### Oxidative stress response

The present study showed that genes encoding for antioxidant enzymes, such as peroxiredoxin 2 (PRDX2), PRDX3 and thioredoxin 2 (TXN2) were found to be up-regulated by U, indicating possible activation of antioxidant defense system. It has been commonly accepted that U may generate ROS and/or interfere with cellular redox reactions similar to other metals, such nickel, cadmium and copper [31], and subsequently cause oxidative stress as a key MoA [7, 15, 16, 19, 32]. Previous work by Song et al. [7] also documented the up-regulation of several genes involved in the antioxidant system in the liver of salmon after 48 h exposure to U, such as γ-glutamyl cysteine synthetase (GCS), glutathione reductase (GR) and glutathione peroxidase (GPx).

Since ROS can be formed following exposure to exogenous stressors such as U, or through normal biochemical processes in various cellular components such as cell membrane, mitochondria, peroxisomes and endoplasmic reticulum, clear identification of the actual source of ROS production may be challenging based on transcriptional responses. As the elevated level of mitochondrial ETC activity may also be a source of ROS production through normal physiological processes [33], it was likely that at least part of the oxidative stress response observed may occur in the mitochondria as a result of increased redox activities in the ETC Such hypothesis may also be supported by the identification of only limited number of DEGs related to antioxidant defense in this study. Furthermore, depolarization of mitochondrial membrane has been reported as a protective mechanism to avoid excessive ROS formation in the mitochondria in mammals [34, 35], although such causal relationship has not been confirmed in fish yet.

#### Apoptotic signaling

The swelling of mitochondria is frequently accompanied with apoptosis due to the release of cytochrome c as an initiator. Several DEGs found in this study, such as P53 and AIFM1 supporting the toxicity pathway of mitochondrial swelling, are also key regulators of apoptosis. The activation of apoptotic signaling (Additional file 2: Figure S2) supported by DEGs such as P53, AIFM1, bcl2-related myeloid cell leukemia sequence 1 (MCL1), B-cell lymphoma (Bcl) 2-associated X protein (BAX), apoptotic chromatin condensation inducer 1 (ACIN1) and spectrin alpha non-erythrocytic 1 (Fodrin) that were regulated by low and medium U, strengthened the hypothesis that apoptosis was potentially activated after short-term U exposure. Based on these DEGs, it seemed that three types of apoptotic signaling pathways may be affected, including the intrinsic apoptosis (BAX, P53), extrinsic apoptosis (MCL1) and caspase-independent apoptosis (AIFM1). However, genes regulating the outcomes of apoptotic signaling, such as ACIN1 promoted chromosome condensation and fodrin-regulated cell shrinkage and membrane blebbing were indeed repressed, probably indicating strict regulation of programmed cell death by other physiological processes as well. A previous study by the current research group [7] showed that caspase family genes such as BAX, Bcl-x and Caspase 6A were significantly up-regulated as an early sign of apoptosis in Atlantic salmon after 48 h exposure to U. Lerebours et al. [6] found that the BAX gene was 4-fold (23 μg U/L) and 10-fold (130 μg U/L) up-regulated in the liver of zebrafish after a 28 d exposure, suggesting that U exposure may cause apoptosis in fish at even lower concentrations than those tested herein.

#### Hypoxia signaling

The hypoxia-inducible factor (HIF) signaling pathway (Additional file 2: Figure S3) supported by DEGs such as P4HB, P53, HSP90AB1, NAD(P)H dehydrogenase quinone 1 (NQO1), ubiquitin-conjugating enzyme E2E 1 (UBE2E1) and SMT3 suppressor of mif two 3 homolog 1 (SUMO1) was found to be affected by all U concentrations. Key DEGs in this pathway promoting the hydroxylation (P4HB), ubiquitination (P53, HSP90AB1, NQO1, UBE2) and transcriptional regulation (SUMO1) of HIF-α were induced by U, suggesting that salmon may have suffered from U-caused hypoxia [36–42]. Although not assessed in the present study, the rationale for these observations may be several. Firstly, fish may have actually experienced hypoxia, as U may accumulate in the gill [6, 7, 43], alter gill structure and functions to reduce the gill oxygen uptake [43], influence the capacity of oxygen transport by red blood cells, or reduce the cardiac flow rate and oxygen supply [44]. The hypoxic responses have been frequently observed in living organisms exposed to metals, such as chromium, nickel and cobalt [45]. Secondly, dysfunction of the hematological system, which was indeed found to be one of the top gene networks regulated by all U concentrations, may potentially lead to hypoxia by affecting the transportation of oxygen. Hypoxic stress may cause further damage to the cardiovascular system and/or other physiological processes [46]. Another potential mechanism may be that cross-talks between pathways such as P53 signaling and/or aryl hydrocarbon receptor (AhR) pathway may cause modulation of HIF-α signaling [47]. In addition, U-induced ROS may also interfere with the iron availability and regulate the prolyl hydroxylase activity indirectly [48, 49]. Since 95% of the oxygen consumed by fish is used by the mitochondrial ETC to produce ATP, hypoxia may have considerable impact on the ETC activity. Several studies have proposed that mitochondrial ETC may regulate the cellular ROS level and HIF-1α expression [50], supporting the hypothesis that the induction of hypoxia signaling may also be a consequence of mitochondrial dysfunction.

#### DNA repair signaling

Uranium has been reported to cause DNA damage in fish [15, 19] either by causing oxidative DNA strand breaks through ROS, or by direct alteration of DNA structure through binding to the DNA molecules and forming uranium-DNA adducts [51, 52]. The top network found in this study showed that U regulated a group of DEGs associated with DNA replication, recombination and repair, representing transcriptional responses to potential DNA damage caused by U. Although no toxicity pathway directly associated with DNA damage was found based on the current data, the significant enrichment of a canonical pathway related to nucleotide excision repair (NER) may provide a link between U exposure and DNA damage. The NER pathway is usually responsible for repairing single strand DNA damage, either by global genome NER (GG-NER), or transcriptionally coupled NER (TC-NER). The present study identified up-regulated DEGs involved in both types of NER, such as DNA directed RNA polymerase II polypeptide I (POLR2I), DNA directed RNA polymerase II polypeptide F (POLR2F) and general transcription factor II H polypeptide 1 (GTF2H1) which are part of the TC-NER pathway. The nuclear excision repair protein RAD23 homolog B (RAD23B), which plays a central role in the GG-NER pathway, were also found to be affected by exposure to U. Song et al. [7] has previously observed that multiple genes involved in the cell cycle regulation and DNA repair processes, such as P53, cyclin-dependent kinase inhibitor 1 (P21), growth arrest and DNA damage-inducible gene gamma (GADD45G), proliferating cell nuclear antigen (PCNA) and RAD51, were up-regulated in the liver of Atlantic salmon after 48 h exposure to U. These evidences suggested that U may cause DNA damage after short-term exposure. But these damages may also be rapidly repaired by various DNA repair mechanisms.

#### Regulation of immune responses

Uranium has been documented to induce immune responses in fish. Cooley and co-workers [53] observed a wide range of histological changes, such as inflammation, tubules necrosis, haemorrhaging, glomerular lesions, pigmented macrophage proliferation in the liver of lake whitefish (*Coregonus clupeaformis*) after dietary exposure to U. A study on zebrafish [6] showed that hepatic IL-1b gene was 4-fold up-regulated by 23 μg /L U after 28 d waterborne exposure and 45-fold up-regulated by 130 μg /L U after 28 d exposure, suggesting that U may induce inflammatory responses. Gagnaire et al. [54] measured the immune biomarker enzyme phenoloxidase-like (PO) activity in zebrafish and found increased PO activity in adult fish after 48 h exposure to U, but significantly decreased PO activity in 96 h larvae after 4 d exposure to U. In mammals, Taulen and colleagues [55] found a group of genes associated with immune functions including tumor necrosis factor alpha-induced protein 1 (TNFAIP1) to be up-regulated in mouse kidney after a 48 h intraperitoneal injection exposure of 5 mg/kg uranyl nitrate. An *in vitro* gene expression study on murine macrophages and CD4+ T-cells showed that U induced multiple genes related to signal transduction, neurotrophic factors, chemokine and chemokine receptors, and interleukins, suggesting the immune modulation ability of U [56].

Several canonical pathways were found to be associated with U-mediated immune responses in this study. The antigen presentation pathway, which plays an important role in the development of both innate and adaptive immunity, may be one of the key pathways linking U exposure to the initiation of immune responses. It is well-known that T helper (CD4+) cells are vital in assisting other white blood cells in immunologic processes, whereas cytotoxic (CD8+) T-cells are eliminators of problematic cells. Through the antigen presentation pathway, cell types such as macrophages and dendritic cells are able to capture antigens and recognized by CD4+ and CD8+ T-cells. A few central DEGs in this pathway, such as major histocompatibility complex class II DQ beta 2 (HLA-DQB2), major histocompatibility complex class I C (HLA-C) and ATP-binding cassette sub-family B transporter 1 (TAP1) were found to be up-regulated following U exposure. The major histocompatibility complexes (MHCs) are often considered to be the main functioning molecules for immune recognition. The TAP1 gene encodes protein for transporting fragmented peptides during antigen presentation. The effect of U on antigen presentation has not been well studied yet, but the toxicological mechanisms may be similar to that proposed for nickel [57], including indirect actions to self-proteins which are then processed and presented by MHC, or directly binding to MHC/peptide complexes. Other immune-related canonical pathways, such as the IL-22 and HMGB1 signaling, which are involved in inflammation as an innate immune response [58], were found to be commonly regulated by all U concentrations. Another canonical pathway, the IL-8 signaling, which is central to inflammation, angiogenesis and tumor growth processes, was found to be regulated by low and medium U treatments. However, as the key regulator genes in these pathways such as IL-22, HMGB1 and IL-8 were not identified as DEGs in the current study, it was not clear how these pathways were affected by U.

#### Other potential mechanisms

Besides the major MoAs identified, the global transcriptional analysis performed herein may also provide some insight into other potential toxicological mechanisms of U which have not been well-studied. Although not as clearly supported by either experimental evidences or previous studies in fish, interference with nuclear receptors (NRs), interaction with peripheral nervous system (PNS) and disturbance of blood coagulation may also be important biological processes that were affected by U in the present study.

Pathways related to the glucocorticoid receptor signaling and retinoid X receptors (RXR) functions such as lipopolysaccharide (LPS)/IL-1 mediated inhibition of RXR function and farnesoid X receptor (FXR)/RXR activation were found to be affected by U. These pathways are important in the transcriptional regulation through NRs and may influence many downstream processes such as regulation of endocrine system, transportation, enzyme metabolism and biosynthetic processes. As DEGs identified in this study only supported the downstream parts of these pathways, it was not clear whether the NR signaling was directly or indirectly affected by U.

Another pathway of potential toxicological interest may be the γ-aminobutyric acid (GABA) receptor signaling regulated by all U concentrations. Supporting DEGs, including two main neurotransmitter receptors in this pathway, GABA A receptor rho 1 (GABRR1) and GABA A receptor beta 2 (GABRB4), were up-regulated after 48 h exposure. The GABA signaling is usually present in the central nervous system (CNS), but also widely found in peripheral tissues, including fish liver [59]. The non-CNS roles of GABA in fish have been considered to participate in the regulation of reproduction, such as enhancing the release of gonadotropins from the pituitary or altering the plasma luteinizing hormone level [60]. It has been proposed for mammals that the activation of GABA receptors may reduce the oxidative damage to the liver [61]. Since no fish studies have been performed so far on U-induced GABA signaling in peripheral tissues, it may only be speculated that the induction of GABA signaling may be a secondary effect of oxidative stress or altered calcium level, as these primary effects may interfere with the neurotransmitter receptor signaling and/or inter-cellular signal transductions.

Uranium may also affect the blood coagulation in fish, as the canonical pathway related to the coagulation system was found to be among the most affected pathways by all U concentrations. Key DEGs such as coagulation factor VII (F7), IX (F9) and X (F10) were found to be up-regulated following short-term U exposure. The coagulation system may cross-talk with the immune system, as by forming blood clots, coagulation may physically trap the invading substances. It was also reported that metal-induced hydroxyl radicals may affect the blood coagulation in human [62]. Whether this is also the case in fish still needs to be further investigated.

### Mechanisms of uranium hepatotoxicity

**Figure 9:**
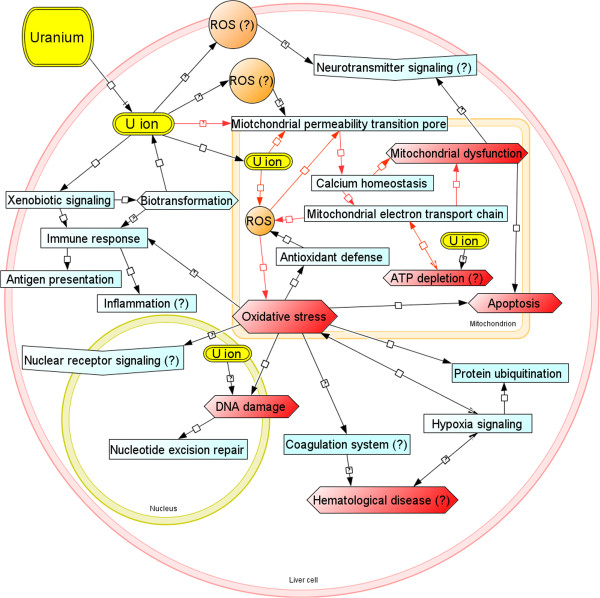
**Putative toxicological mechanisms of depleted uranium (DU).** Proposed network of early toxicological mechanisms of DU hepatotoxicity in Atlantic salmon (*Salmo salar*) after short term (48 h) waterborne exposure. Yellow tags indicate primary stressor; Orange tags indicate secondary stressor; red tags indicate main molecular events or apical endpoints; red lines indicate key mechanisms of action; blue tags indicate key pathways.

A putative network describing the early hepatic toxicological mechanisms of U in Atlantic salmon is proposed in Figure 9. The results from the present study suggested that ionic U species (e.g. uranyl) may accumulate in the liver and exert toxicity mainly through two potential MoAs, one was the induction of organellar or cellular-wide oxidative stress, the other was the uncoupling of OXPHOS in the mitochondrion. The former MoA has been widely accepted, as U may interfere with cellular redox reactions and induce ROS. The latter one has not been as extensively studied, but may represent an important aspect of U hepatotoxicity. The mitochondrion is key for successful energy production and plays important roles in various biological processes such as apoptosis, antioxidant defense and DNA repair. Perturbation of mitochondrial functions caused by U may be contributed by several potential mechanisms, including alteration of membrane permeability, disruption of proton gradient across the inner membrane, dissipation of transmembrane potential or direct binding to phosphates (e.g. ATP and ADP) and subsequently disturbing the synthesis and translocation of ATP. These actions may ultimately lead to decreased cellular energy supply. Both MoAs are considered to stimulate or suppress the mitochondrial ETC activity, depending on the level and type of perturbation. Based on the current experimental evidences, it is plausible that the mitochondrial ETC activity was elevated to compensate for potential loss of MMP and ATP as a compensatory mechanism. Furthermore, it is more likely that the relatively low level of oxidative stress induced by U based on the antioxidant gene responses found in this study may not only be a direct effect of U, but also be contributed by the elevated ROS production by ETC. Several key processes may take place following the molecular initiating events after short-term U exposure, such as biotransformation, immune responses, apoptotic signaling, DNA repair, hypoxia signaling and macromolecule degradation as a consequence of oxidative damage and/or mitochondrial dysfunction. Uranium may also affect the NR signaling, somatic neurotransmitter signaling and blood functions (e.g. coagulation system), which were likely secondary effects following major MoAs of U.

## Conclusion

The present study performed a short-term (48 h) waterborne exposure of Atlantic salmon to three concentrations (0.25, 0.5 and 1.0 mg/L) of DU with the aim to understand the early toxicological mechanisms of U based on global transcriptional changes. Apparent bell-shaped concentration-dependent responses were observed in both global (microarray) and single (qPCR) gene expression analyses, although a number of key responses had already taken place at the lowest U concentration (0.25 mg U/L). The mitochondrion was likely the main target of U after short-term exposure. The induction of oxidative stress and uncoupling of OXPHOS were proposed as two potential MoAs of U at all test concentrations in this study. Other responses, such as the activation of apoptosis signaling, DNA damage and repair, immune response, nuclear receptor signaling, neurotransmitter signaling and haematological response were also considered to be induced by U. Due to the short exposure duration in the current study, no successful phenotypic anchoring was achieved to link molecular responses to adversity at higher organismal levels. However, results obtained herein were in good accordance with previously published U studies in which adverse effects were observed in fish at even lower concentrations of U but longer exposure durations, suggesting that low-dose chronic effects of U should be focused in the future research, in order to link U exposure to adverse effects on basis of the adverse outcome pathway (AOP) concept. This paper has for the first time systematically documented the early stress responses in Atlantic salmon after short-term exposure to environmentally relevant concentrations of DU and may provide substantial mechanistic knowledge for future ecological hazard and risk assessment of environmental radionuclides.

## Methods

### Exposure and sampling

Juvenile (parr) of Atlantic salmon (length 10.6 ± 0.6 cm, weight 11.4 ± 2.3 g) from the Fish Laboratory of Norwegian University of Life Sciences (NMBU, Ås, Norway) was acclimated to the experimental water conditions at the Isotope Laboratory of NMBU (4.5°C, pH 7.1, December 2010). Feeding of test fish was terminated 48 h prior to transportation of fish to the Isotope Laboratory.

For U exposure, six test fish for each treatment group were transferred to lake water (Maridalsvannet, Norway) for 72 h acclimation. Test fish were then exposed to 0 mg/L (control), 0.25 mg/L, 0.5 mg/L and 1.0 mg/L dissolved uranyl acetate dihydrate (UO_2_(CH_3_COO)_2_ · 2H_2_O, purity ≥ 98.0%, specific activity 1.459 × 10^4^ Bq/g, 57.3% of the radioactivity from natural U, obtained from Fluka, Sigma-Aldrich, Buchs, Switzerland) for 48 h in the dark. The experiment was conducted in a static exposure system (25 L tank, fish loading approx. 2.7 g/L) served with air pumps. Immediately after the exposure, fish were checked for potential morphological changes, including alterations of skin color, mucus quality, gill shape and gill color. Fish were then sacrificed by cephalic concussion and dissected to collect tissue samples. Liver samples for gene expression analysis were snap-frozen in liquid nitrogen and stored at −80°C. Blood samples were also collected to determine the plasma glucose, ions (Na, K) and hematocrit levels using an i-STAT® portable analyzer (Abbott Point of Care Inc., Princeton, NJ, USA) with EC8+ cassette (Abbot, East Windsor, USA), and to assess the chromosomal damage using micronucleus assay [7]. Exposure conditions (e.g. pH, temperature) and water variables (e.g. conductivity, total organic carbon, major cations and anions) were measured throughout the experiment. Uranium concentrations in water were determined before and after the exposure. The liver concentrations of U were also measured after the exposure. For more detailed method descriptions of exposure and chemical analysis, please consult an earlier publication on this exposure experiment [7] by current research group.

All treatments of fish were in accordance with the Norwegian Welfare Act and research animal legislation, and the experiment was approved in advance (30-11-2010) by the local representative of the Norwegian Animal Research Authority (NARA ID: 3026).

### RNA isolation

Total RNA was isolated using RNeasy Plus Mini kit (Qiagen, Hilden, Germany) according to the manufacturer’s instructions. Briefly, approximately 25 mg of snap-frozen liver sample was lysed in 600 μL buffer RLT plus™ containing β-mercaptoethanol (1% v/v) (Sigma-Aldrich Chemie, Steinheim, Germany). The lysate was homogeinized (3 × 10 sec, 6000 rpm) with Precellys CK14 beads using a Precellys orbital shaker bead mill (Bertin, Montigny-le-Bretonneux, France). After homogenization, samples were incubated for 5 min and centrifuged (12000 g) for 3 min (20°C) to separate the remaining tissue debris. The clear supernatants were transferred to the gDNA EliminatorTM spin column (Qiagen) and centrifuged (8000 g, 30s) to remove genomic DNA. The eluate was then mixed well with 350 μL ethanol (50%) and transferred to RNeasy spin column (Qiagen). The columns were centrifuged (8000 g) for 15 s, washed once with 500 μL buffer RW1, centrifuged once at 8000 g (15 s) then washed 2 times with 500 μL buffer RPE and finally centrifuged at 8000 g (15 s and 2 min, respectively). Thirty μL of nuclease-free water was added to the column and the column was centrifuged (8000 g, 1 min) to obtain a pure RNA eluate.

The RNA quality (purity and yield) was controlled by photometric analyses (260/230 > 2.0, 260/280 > 1.8, yield > 200 ng/μL) using Nanodrop® spectrophotometer (ND-1000, Nanodrop Technologies, Wilminton, Delaware, USA) and RNA integrity (RIN > 9.0) inspected by Bioanalyzer gel electrophoresis with RNA 6000 Nano chips (Agilent technologies, Santa Clara, California, USA) according to the manufacturer’s instructions. The RNA samples were stored at −80°C for further analyses. Three RNA samples per treatment group were used for microarray analysis and all six samples per treatment were used for qPCR analysis.

### Microarray analysis

The 60,000-feature (60 k) high density custom salmonid oligonucleotide microarray was manufactured by Agilent Technologies (Santa Clara, CA, USA). The microarray probes were designed based on Atlantic salmon (*Salmo salar*) and Rainbow trout (*Oncorhynchus mykiss*) consensus sequences (contigs) and single ESTs from the cGRASP project [63] and Unigene [64] for the two species (*Salmo salar*: build 31 and *Oncorhynchus mykiss*: build 27). The microarray platform can be accessed at Gene Expression Omnibus (GEO, accession number: GPL18864).

The microarray gene expression analysis (N = 3) was performed according to Agilent’s standard protocol “One-Color Microarray-Based Gene Expression Analysis, version 6.5”. Except for acetonitrile (purity ≥99.5%, Sigma-Aldrich, St. Louis, MO, USA), all reagents for microarray analysis were purchased from Agilent. Briefly, 200 ng of total RNA (in 1.5 uL nuclease-free water) from each sample was mixed with spike-in standard (Agilent One-Color Spike Mix Kit) for later verification of the dynamic range and linearity of fluorescence signal. A T7 promotor primer (Agilent Low Input Quick Amp Labeling Kit) was then added and both RNA template and T7 primer were denatured at 65°C for exact 10 min before being cooled rapidly on ice for 5 min. First strand cDNA was synthesized by incubating the template with 5× First Strand Buffer, 0.1 M dithiotreitol (DTT), 10 mM deoxyribose nucleotide mixture (dNTP mix) and AffinityScript RNase Block Mix (Agilent Low Input Quick Amp Labeling Kit) at 40°C for 2 h. The cDNA and enzymes were then denatured by incubating at 70°C for 15 min and rapidly cooled on ice for 5 min. The cRNA was synthesized by incubating cDNA samples with 5× Transcription Buffer, 0.1 M DTT, NTP mix, Cyanine 3-CTP (Cy3) and T7 RNA Polymerase Blend (Agilent Low Input Quick Amp Labeling Kit) at 40°C for 2 h and purified using Qiagen RNeasy Mini spin columns as recommended in the protocol. The eluate was quality checked by nanodrop to determine the cRNA yield (>0.825 μg) and dye specific activity (>6 pmol Cy3 per μg cRNA). Then, 600 ng of cy3-labeled, linearly amplified cRNA was fragmented by incubating with 10× Blocking Agent and 25× Fragmentation Buffer (Agilent Gene Expression Hybridization Kit) at 60°C. The fragmentation reaction was stopped immediately after exact 30 min by cooling the cRNA samples on ice for 1 min., cRNA from the Fragmentation Mix was then mixed well with the same volume of 2× GEx Hybridization Buffer HI-RPM (Agilent Gene Expression Hybridization Kit), spun down at 13,000 g for 1 min, and carefully pipette onto the gasket slides and assembled with the microarray slide. All slides were rotationally (10 rpm) incubated at 65°C for 17 h. After hybridization, the slide was disassembled in Agilent Gene Expression Wash Buffer 1 (containing 0.005% v/v Triton X-102), washed once with Agilent Gene Expression Wash Buffer 1 at room temperature and washed for another round with Agilent Gene Expression Wash Buffer 2 (containing 0.005% v/v Triton X-102) at approx. 31°C. The array slide was then dried by using acetonitrile (Sigma-Aldrich, St. Louis, MO, USA) and immediately scanned in an Agilent C scanner (Agilent Technologies) using a scan region of 61 × 21.6 mm, resolution of 3 μm and output Tiff image of 20 bit.

### Quantitative real-time rtPCR

The representativeness of microarray probe hybridization and specific gene expression responses of toxicological interests were verified using qPCR (N = 6). Briefly, total RNA (2 μg) was reversely transcribed with random hexamer priming, using a High Capacity cDNA Archive Kit (Applied Biosystems, Foster City, California, USA) according to the manufacturer’s instructions. Gene expressions were then assayed in an absolute quantification protocol by real-time PCR on a Bio-Rad CFX 384 (Bio-Rad Laboratories, Hercules, CA, USA) platform. Primer sequences (Table 3) were designed using Primer3 v0.4.0 software [65], based on the consensus sequences of microarray probe contig sequences and putative mRNA/EST sequences in Genbank [66] and Unigene [64]. All primers were purchased from Invitrogen™ (Carlsbad, California, USA) and optimized for annealing temperature and amplification efficiency. The qPCR reactions were then run in technical duplicates with total volume of 10 μL each, and contained cDNA template made from 10 ng of RNA, PerfeCTa® SYBR® Green FastMix® (Quanta BioSciencesTM, Gaithersburg, MD, USA) and 400 nM forward/reverse primer. The qPCR conditions were 95°C for 3 min, 40 cycles of 95°C for 20 s, primer-specific annealing temperature for 20 s and 72°C for 30 s, and finally followed by a melting curve determination step. The amplification was considered to be valid if only one unique product peak was identified by melting curve analysis. Non-template controls (NTCs) and no-reverse-transcriptase controls (NRTs) were included as quality assurance to identify potential DNA contamination. Standard curves were run with 50, 10, 2 and 0.4 ng of pooled cDNA from all samples, and relative expression was then determined from the standard curves based on threshold cycle (Cq) value and absolute efficiency values using the ΔCq method implemented in the Bio-Rad CFX Manager v2.0 software. Results were normalized to the geometric mean of reference gene expression using the ΔΔCq method in CFX Manager (Bio-Rad), and compared to that of the control for calculation of absolute fold changes (FCs). The 18S ribosomal RNA (18S) and ribosomal protein L1 (RPL1) were used as reference genes due to their stable expression irrespective of different treatments.

**Table 3:** **Primer sequences for quantitative real-time rtPCR of selected potential biomarker genes**

Target gene	Genbank accession	Forward (5’-3’)	Reverse (5’-3’)	Product size (bp)	Efficiency (%)	Annealing temp. (°C)
AIFM1	JT833124	CCCTACTGGCATCAGTCCAT	TGTCCTTCTGCTCGGGTACT	250	96.8	59.1
HSP90AB1	NM_001123532	TCATGGACAGCTGTGAGGAG	CCAGCTTGAGGTTCTTGGAG	234	95.5	56
SDHD	BT071922	GCGCATGCACTTAGTCAAAA	GGTGCATTTTTCTTCCCAAA	248	90.8	59.1
P4HB	BT072340	TAAGCGTGATTGCGTGAGTC	TGTGATGGAATGCGTTTGTT	175	95.7	56
COX6B1	BT125515	GACAATGCTTGGCACATACG	TGTCAGCAGATGCAGAGTCC	218	95.4	59.1
PRDX3	BT046676	CTAAGTGGGCTCCAGCTGTC	ATGATCTCTGTCGGGCAAAC	163	96.4	56
P53	EZ772237	GAGGAGATCAACCTGAAGAAGCA	AGGCCTCCTTCATAGCACGTT	91	100	63.4
JAK1	BT057852.1	ACTAACTGGCATGGGACCAG	CCAGACCCTTCTGGAAATCA	172	95.2	59.1
18S	AJ427629	TGTGCCGCTAGAGGTGAAATT	GCAAATGCTTTCGCTTTCG	61	95.4	59.1
RPL1	CB516726	ACTATGGCTGTCGAGAAGGTGCT	TGTACTCGAACAGTCGTGGGTCA	118	95.3	60

Full descriptions of gene symbols can be found in Additional file 1: Table S3.

### Bioinformatics and biostatistics

Scanned microarray images were extracted using Agilent Feature Extraction software v10.7. Raw microarray data has been deposited at GEO (accession number: GSE58824). Data normalization and statistical analyses were performed using GeneSpring GX v11.0 (Agilent Technologies). Differentially expressed genes (DEGs) were determined using one-way analysis of variance (ANOVA) followed by Benjamini and Hochberg (BH) False Discovery Rate (FDR) correction (corrected p-value <0.5). Fold change cut-off of 1.5 was applied to all downstream analyses. To identify major patterns of global transcriptional changes, a K-means clustering analysis was performed using R-3.0.2 [67] according to the method described elsewhere [68]. The number of representative clusters was chosen based on a comparison of difference between the actual and random sum of squared error (SSEs) in the data against different tested cluster solutions. A Tukey honestly significant difference (HSD) posthoc analysis was further performed to identify treatment-related DEGs. Venn diagrams were made using Venny [69].

The functional enrichment analysis of microarray data was performed in Cytoscape v2.8 [70] Bingo v2.4 plugin [71] using a hypergeometric tests with BH FDR correction. The overrepresented GO terms were submitted for GOSlim analysis to reduce redundancy and make directed acyclic GO graphs (DAGs) using BLAST2GO software [72].

The pathway analysis was performed using Ingenuity Pathway Analysis (IPA) [73] with mammalian orthologs from the RefSeq [74] protein database. Orthologs were identified with standalone Inparanoid 4.1. [75] using BLAST 2.2.27+ binaries from NCBI to make the four all-against-all input files. All BLAST searches were made on a protein basis using BLASTx, tBLASTn and tBLASTx to make appropriate translation between nucleotide and protein sequences. As recommended by the developers of Inparanoid [76], a two-pass BLAST approach was applied, using low complexity filter (SEG) and compositional adjustment where available in the first pass, and re-aligning the matches with SEG and compositional adjustment turned off in the second phase. Score threshold was set to 1 bit, and due to the relative short length of the expressed sequence tags (ESTs) and contiguous (contigs), the cut-off values for sequence overlapping and segment coverage in the Inparanoid algorithm were set to 0.001 and 0.0005, respectively.

Statistical analyses of qPCR data were performed using Graphpad Prism v5.0 (Graphpad Software, Inc., San Diego, CA, USA). Normal distributed data with equal variance were subjected directly to statistical analysis whereas data showing unequal variance was log10 transformed prior to assessment of group differences by a one-way ANOVA test followed by a Tukey’s post-hoc tests. A Kruskal-Wallis non-parametric test followed with Dunn’s post-hoc tests was performed for transformed data sets which failed to meet the criteria of equal variance. A probability (p) level of 0.05 was applied to all statistical tests.

## Availability of supporting data

The raw data sets supporting the results of this article are available in the Gene Expression Omnibus (GEO) repository, accession number: GSE58824, http://www.ncbi.nlm.nih.gov/geo/query/acc.cgi?acc=GSE58824.

Other data sets supporting the results of this article are included within the article (and its additional files).

## Electronic supplementary material

Additional file 1: Table S1: Differentially expressed genes (DEGs) that were regulated in the liver of Atlantic salmon (*Salmo salar*) after 48 h waterborne exposure to 0.25, 0.5 and 1.0 mg/L nominal concentrations of depleted uranium (DU). **Table S2.** Overrepresented Gene Ontology (GO) functions that were regulated in the liver of Atlantic salmon (*Salmo salar*) after 48 h waterborne exposure to 0.25, 0.5 and 1.0 mg/L nominal concentrations of depleted uranium (DU). **Table S3.** Mapped Atlantic salmon (*Salmo salar*) differentially expressed genes (DEGs) towards mammlian orthologs. **Table S4.** Toxicity pathways and supporting differentially expressed genes (DEGs) that were regulated in the liver of Atlantic salmon (*Salmo salar*) after 48 h waterborne exposure to 0.25, 0.5 and 1.0 mg/L nominal concentrations of depleted uranium (DU). Full descriptions of gene symbols can be found in Additional file 1: Table S3. Table S5 Canonical pathways and supporting differentially expressed genes (DEGs) that were regulated in the liver of Atlantic salmon (*Salmo salar*) after 48 h waterborne exposure to 0.25, 0.5 and 1.0 mg/L nominal concentrations of depleted uranium (DU). Full descriptions of gene symbols can be found in Additional file 1: Table S3. (XLSX 223 KB)

Additional file 2: Figure S1: Mitochondrial dysfunction. An illustration of pathways associated with mitochondrial dysfunction (modified from Ingenuity Pathway Analysis [73]). Colored components indicate experimental evidences in the present study, orange: up-regulated, green: down-regulated. Full descriptions of gene symbols can be found in Additional file 1: Table S3. **Figure S2.** Apoptosis signaling. An illustration of pathways associated with apoptosis signaling (modified from Ingenuity Pathway Analysis [73]). Colored components indicate experimental evidences in the present study, orange: up-regulated, green: down-regulated. Full descriptions of gene symbols can be found in Additional file 1: Table S3. **Figure S3.** Hypoxia-inducible factor signaling. An illustration of canonical pathway associated with hypoxia-inducible factor signaling (derived from Ingenuity Pathway Analysis [73]). Colored components indicate experimental evidences in the present study, orange: up-regulated. Full descriptions of gene symbols can be found in Additional file 1: Table S3. (DOCX 769 KB)
