# Supplementary material for: Hepatic transcriptomic profiling reveals early toxicological mechanisms of uranium in Atlantic salmon (Salmo salar)
Source: BMC Genomics. 2014 Aug 20;15(1):694. doi: 10.1186/1471-2164-15-694 (PMC4148957; doi:10.1186/1471-2164-15-694)
Supplement: Supplementary file 2 — Additional file 2: Figure S1: Mitochondrial dysfunction. An illustration of pathways associated with mitochondrial dysfunction (modified from Ingenuity Pathway Analysis [73]). Colored components indicate experimental evidences in the present study, orange: up-regulated, green: down-regulated. Full descriptions of gene symbols can be found in Additional file 1: Table S3. Figure S2. Apoptosis signaling. An illustration of pathways associated with apoptosis signaling (modified from Ingenuity Pathway Analysis [73]). Colored components indicate experimental evidences in the present study, orange: up-regulated, green: down-regulated. Full descriptions of gene symbols can be found in Additional file 1: Table S3. Figure S3. Hypoxia-inducible factor signaling. An illustration of canonical pathway associated with hypoxia-inducible factor signaling (derived from Ingenuity Pathway Analysis [73]). Colored components indicate experimental evidences in the present study, orange: up-regulated. Full descriptions of gene symbols can be found in Additional file 1: Table S3. (DOCX 769 KB) [file 12864_2013_6382_MOESM2_ESM.docx]

# Additional file 2

# Hepatic transcriptomic profiling reveals early toxicological mechanisms of uranium in Atlantic salmon (*Salmo salar*)

**You Song^1,2,*^, Brit Salbu^1^, Hans-Christian Teien^1^, Lene Sørlie Heier^1^, Bjørn Olav Rosseland^1,3^, Tore Høgåsen^2^, Knut Erik Tollefsen^1,2^**

***Correspondence: [you.song@niva.no](mailto:you.song@niva.no)

^1^ Norwegian University of Life Sciences (NMBU), Faculty of Environmental Science and Technology, Department of Environmental Sciences (IMV), Centre for Environmental Radioactivity (CERAD). P.O. Box 5003, N-1432 Ås, Norway

^2^ Norwegian Institute for Water Research (NIVA), Gaustadalléen 21, N-0349 Oslo, Norway

# of pages: 4

# of figures: 3


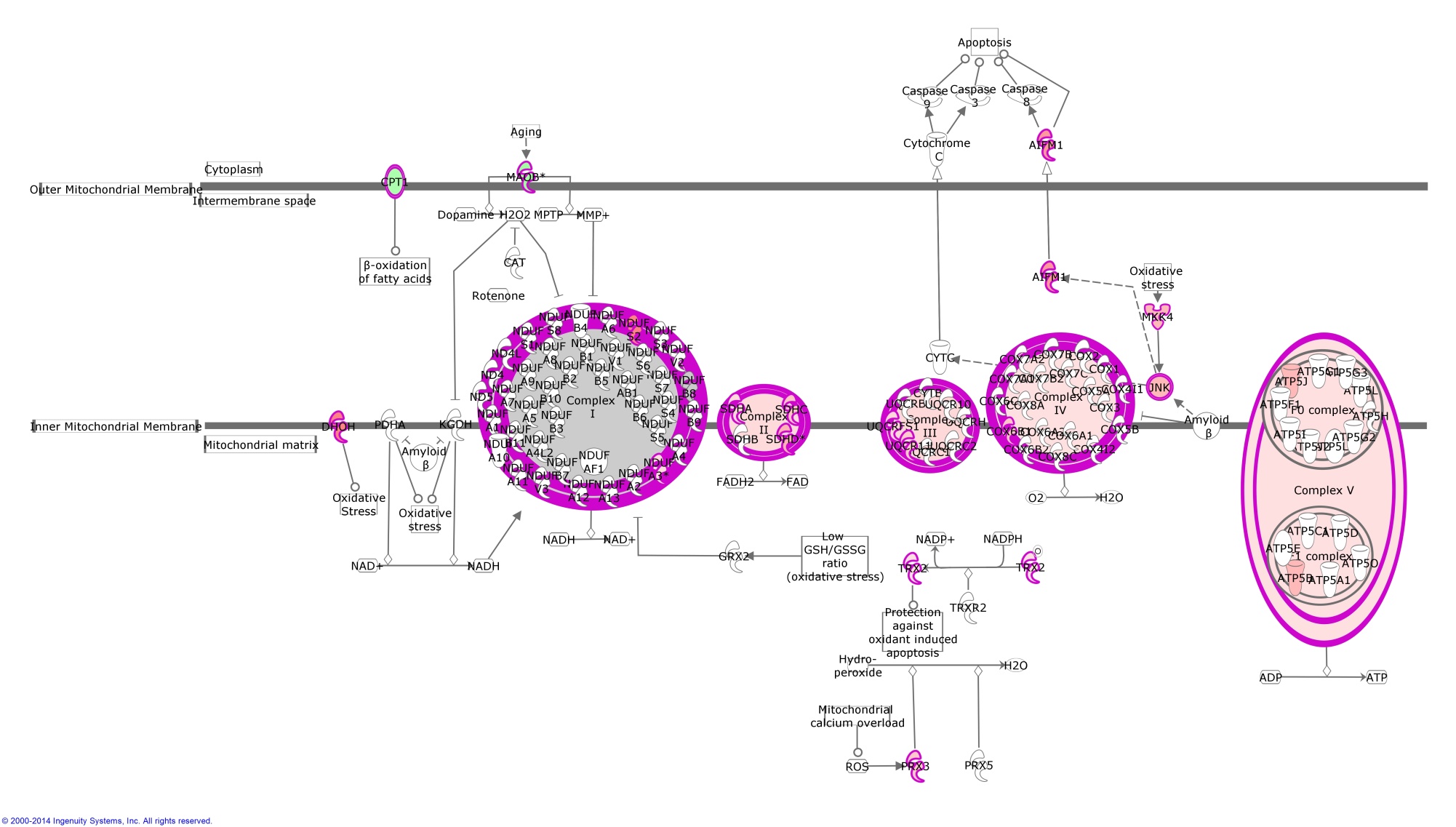


**Figure S1 Mitochondrial dysfunction.** An illustration of pathways associated with mitochondrial dysfunction (modified from Ingenuity Pathway Analysis [[74](#_ENREF_74)]). Colored components indicate experimental evidences in the present study, orange: up-regulated, green: down-regulated. Full descriptions of gene symbols can be found in Additional file 1 (Table S3).


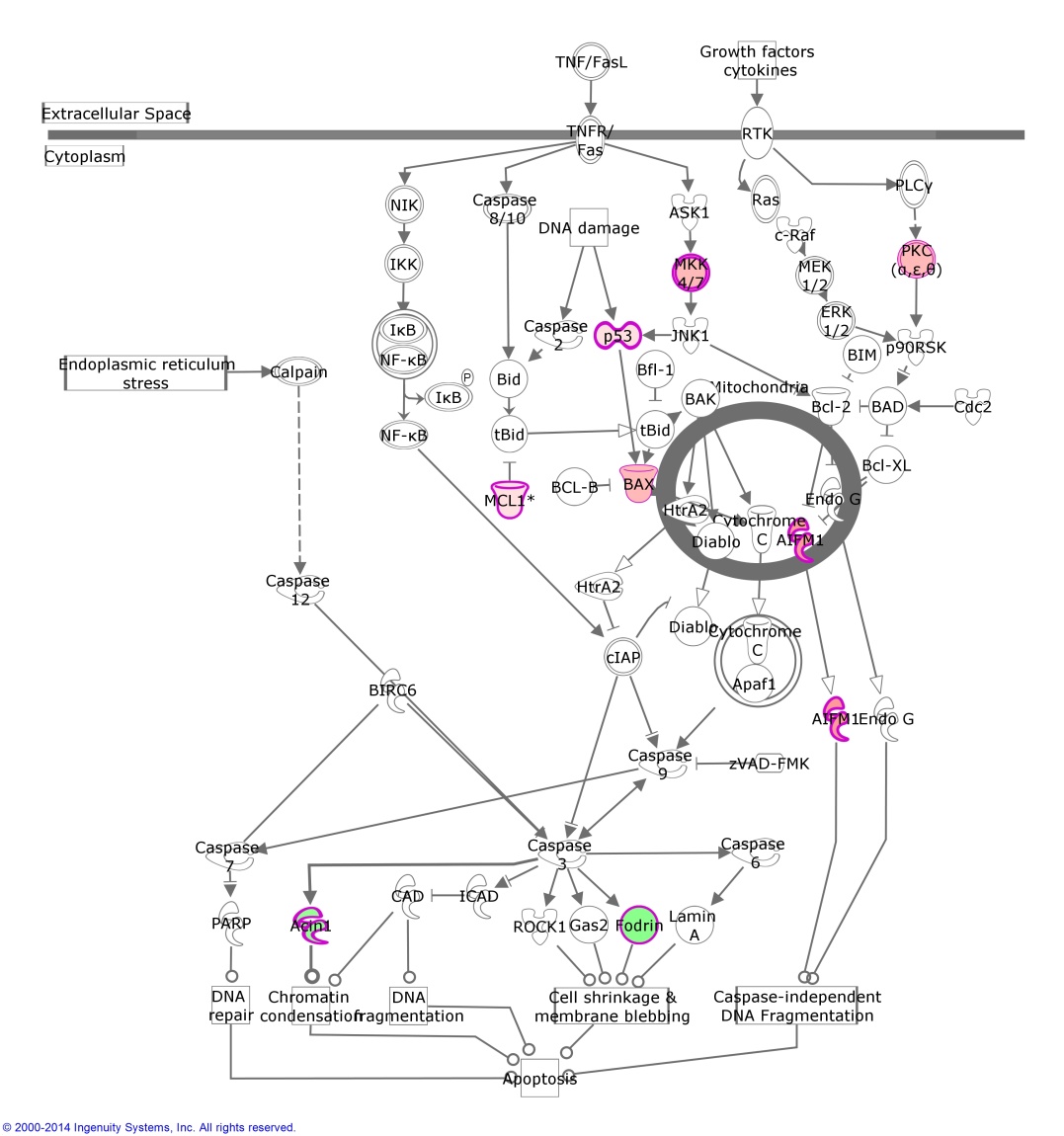


**Figure S2 Apoptosis signaling.** An illustration of pathways associated with apoptosis signaling (modified from Ingenuity Pathway Analysis [[74](#_ENREF_74)]). Colored components indicate experimental evidences in the present study, orange: up-regulated, green: down-regulated. Full descriptions of gene symbols can be found in Additional file 1 (Table S3).


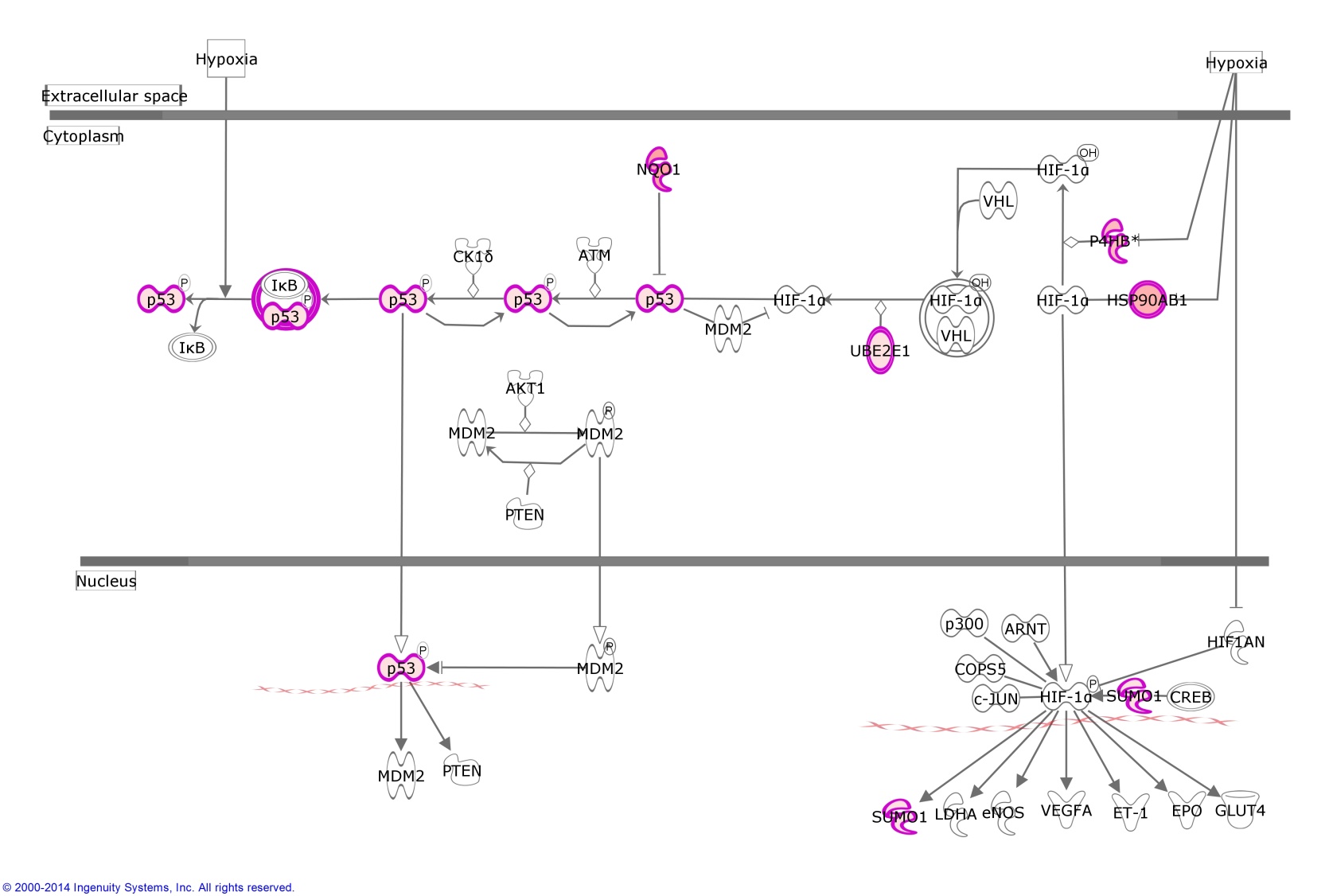


**Figure S3 Hypoxia-inducible factor signaling.** An illustration of canonical pathway associated with hypoxia-inducible factor signaling (derived from Ingenuity Pathway Analysis [[74](#_ENREF_74)]). Colored components indicate experimental evidences in the present study, orange: up-regulated. Full descriptions of gene symbols can be found in Additional file 1 (Table S3).
